# Anti-Retroviral Therapy Increases the Prevalence of Dyslipidemia in South African HIV-Infected Patients

**DOI:** 10.1371/journal.pone.0151911

**Published:** 2016-03-17

**Authors:** Joel A. Dave, Naomi S. Levitt, Ian L. Ross, Miguel Lacerda, Gary Maartens, Dirk Blom

**Affiliations:** 1 Divisions of Diabetic Medicine and Endocrinology, Department of Medicine, University of Cape Town, Cape Town, South Africa; 2 Department of Statistical Sciences, University of Cape Town, Cape Town, South Africa; 3 Division of Clinical Pharmacology, Department of Medicine, University of Cape Town, Cape Town, South Africa; 4 Division of Lipidology, Department of Medicine, University of Cape Town, Cape Town, South Africa; University of Cincinnati College of Medicine, UNITED STATES

## Abstract

**Purpose:**

Data on the prevalence of dyslipidaemia and associated risk factors in HIV-infected patients from sub-Saharan Africa is sparse. We performed a cross-sectional analysis in a cohort of HIV-infected South African adults.

**Methods:**

We studied HIV-infected patients who were either antiretroviral therapy (ART)-naive or receiving non-nucleoside reverse transcriptase inhibitor (NNRTI)-based or protease inhibitor (PI)-based ART. Evaluation included fasting lipograms, oral glucose tolerance tests and clinical anthropometry. Dyslipidemia was defined using the NCEP ATPIII guidelines.

**Results:**

The median age of the participants was 34 years (range 19–68 years) and 78% were women. The prevalence of dyslipidemia in 406 ART-naive and 551 participants on ART was 90.0% and 85%, respectively. Low HDL-cholesterol (HDLC) was the most common abnormality [290/406 (71%) ART-naïve and 237/551 (43%) ART- participants]. Participants on ART had higher triglycerides (TG), total cholesterol (TC), LDL-cholesterol (LDLC) and HDLC than the ART-naïve group. Severe dyslipidaemia, (LDLC> 4.9 mmol/L or TG >5.0 mmol/L) was present in <5% of participants. In multivariate analyses there were complex associations between age, gender, type and duration of ART and body composition and LDLC, HDLC and TG, which differed between ART-naïve and ART-participants.

**Conclusion:**

Participants on ART had higher TG, TC, LDLC and HDLC than those who were ART-naïve but severe lipid abnormalities requiring evaluation and treatment were uncommon.

## Introduction

Timely initiation of antiretroviral therapy (ART) in patients with HIV infection markedly reduces morbidity and mortality.[1–3] However, peripheral lipoatrophy, dysglycemia, dyslipidemia and increased cardiovascular risk[4, 5] are significant concerns with long-term ART.[6–8] Dyslipidemia in patients receiving ART is often characterized by elevation of total cholesterol (TC), low-density lipoprotein cholesterol (LDLC) and triglycerides, with minor changes of high-density lipoprotein cholesterol (HDLC). Antiretroviral drugs implicated in the genesis of dyslipidemia include protease inhibitors (PIs), stavudine, zidovudine, and the non-nucleoside reverse transcriptase inhibitor (NNRTI) efavirenz.[9–12]

Most studies documenting pro-atherogenic dyslipidemia from ART emanate from high-income countries. These data cannot be extrapolated to low- and middle-income countries, which bear the largest HIV burden, as the patient populations studied and ART regimens prescribed differ substantially. Although CD4 counts at ART initiation are increasing in low- and middle-income countries, many patients still commence ART at advanced stages of HIV infection with multiple opportunistic infections and poor nutritional status. African HIV-infected patients are also younger and predominantly female, compared to the mainly middle-aged and male patients found in cohorts from high-income countries.[13] In low- and middle-income countries antiretrovirals associated with an increased risk of dyslipidaemia, like zidovudine and stavudine (d4T), are still widely used, although d4T is being phased out. Differences in diet and genetic background may also affect lipid responses to ART.

The aims of this cross-sectional study were to describe lipid and lipoprotein abnormalities in adult HIV infected patients, who were either awaiting initiation of ART or had received ART for at least six months, to explore associations between lipid variables, glycemic status and body composition.

## Methods

The Metabolic Complications in Highly Active Antiretroviral Therapy (McHAART) study is a cross-sectional, comparative study in HIV-infected participants in whom ART had not yet been initiated (ART-naïve) and participants who had received ART for at least six months. The evaluation of insulin sensitivity and glucose metabolism has been published previously.[13] The study protocol was approved by the Human Research Ethics Committee of the University of Cape Town and all study participants provided written informed consent.

### Participants

HIV-infected adults aged ≥18 years who were ART-naïve or receiving first-line ART (d4T, lamivudine (3TC), and efavirenz or nevirapine) were conveniently sampled consecutively from Crossroads Community Health Care Centre in Cape Town. Participants on second-line ART (zidovudine (AZT), didanosine (ddI) and lopinavir-ritonavir (LPV-r)) were recruited from an additional facility (Gugulethu Community Health Care Centre), as there were smaller numbers of patients on second-line therapy at the time of the study. Participants were excluded if they had: 1) been on ART for less than six months, 2) a history of diabetes mellitus or IGT, 3) had an active, acute opportunistic infection, 4) severe diarrhoea (six or more stools/day), 5) tuberculosis within one month of commencing treatment, 6) received glucocorticoid therapy within the past six months, 7) were pregnant or 8) were known to have renal failure.

At the time our study was conducted, criteria for ART initiation in the South African public healthcare system were World Health Organization Stage IV HIV disease or a CD4 count of <200 cells/mm^3^. Patients with virological failure despite adequate adherence to the first line NNRT-based ART regimen were switched to a PI-based second line regimen.

### Testing procedures

The methods have been described in detail previously[13]. In short, consenting patients attended the study visit after fasting overnight. Trained field workers administered a questionnaire to the participants to obtain data on socio-demographic details, smoking, alcohol and current medication. Data on ART, HIV viral load, CD4 counts, opportunistic infections and co-morbid conditions was collected from patient records. Height, weight, waist circumference, waist-hip ratio (WHR) and calf skin fold thickness were measured as previously described.[13]

We recorded blood pressure as the mean of three readings at one minute intervals after patients had been sitting in a quiet environment for at least twenty minutes. Fasting blood samples were collected and a 75 g oral glucose tolerance test (OGTT) was performed with venous blood samples taken at 0, 30 and 120 minutes for the measurement of plasma glucose and serum insulin concentrations.

### Biochemical analysis

Specimens were centrifuged on the day of collection and plasma samples for glucose analysis were stored at -20°C while serum samples were stored at -80°C until analyzed further. Glucose, total cholesterol (TC) and triglycerides were measured by enzymatic colorimetry on the Cobas 6000 autoanalyzer (Roche, Switzerland). HDL cholesterol (HDLC) was measured using a direct method (Roche HDLC3 assay, Switzerland). LDL cholesterol (LDLC) was calculated using the Friedewald formula, provided triglycerides were less than 4.5 mmol/L.[14]

### Classification of dyslipidemia and dysglycemia

We classified lipid abnormalities according to the National Cholesterol Education Program Adult Treatment Panel III (NCEP ATP-III) guidelines.[15] We considered TC, LDLC and triglycerides of > 5.2 mmol/L, >2.5 mmol/L and > 1.7 mmol/L, respectively as elevated and HDLC of < 1.0 mmol/L as low, irrespective of gender. Participants who had at least one abnormal lipid parameter were classified as dyslipidemic. We classified glycemic status following an OGTT, using the American Diabetes Association (ADA) defined categories of normal glucose tolerance, impaired fasting glucose (IFG, fasting plasma glucose 5.6 mmol/L to 6.9 mmol/L), impaired glucose tolerance (IGT, a 2-hour plasma glucose post-OGTT 7.8 to 11.0 mmol/L) and diabetes mellitus (DM, a fasting plasma glucose ≥ 7 mmol/L or a 2-hour plasma glucose value post-OGTT ≥ 11.1 mmol/L).[16]

### Statistical analysis

We were interested in whether the degree of immunosuppression, as measured by CD4 count, influenced lipid markers in ART-naïve participants. We first examined the relationship between the lipid markers, triglycerides and HDLC, and CD4 count without accounting for potential confounders. We then conducted an exploratory analysis to assess whether age, gender, BMI, waist-hip ratio and glycaemic status individually influenced the relationship between the lipid markers and CD4 count. Having established expectations of the likely relationships, we performed separate regressions of the natural logarithms of triglycerides and HDLC against CD4 count controlling for age, gender, BMI, waist-hip ratio and glycaemic status. We began by fitting full models that included all of these potential predictors of triglycerides and HDLC and their interactions with CD4 count. Insignificant interaction effects were then dropped from the model. The main effects that were not statistically significant and which did not contribute to significant interactions with CD4 count were subsequently removed. The resulting models were further refined based on the insights from the initial exploratory analysis. After removing outliers and influential observations, the residuals of the final models were approximately normally distributed and homoscedastic.

For participants on ART, we examined whether lipid markers were associated with body composition, as measured by waist circumference (as a surrogate marker of visceral adiposity) and calf skin fold thickness (as a surrogate marker of subcutaneous fat), and type of ART (PI-based versus NNRTI-based). In our preliminary analyses, we examined the relationships between these variables and triglycerides, HDLC and LDLC, first ignoring confounders and then considering each of the following potential confounders in turn: age, gender, glycaemic status, CD4 count, BMI, waist-hip ratio, duration of exposure to stavudine and whether efavirenz was the only NNRTI ever prescribed. Multiple regression models were then developed, beginning with full models that included all of the potential confounders and their interactions with calf skin fold thickness and ART type. The full models were then simplified as described in the previous paragraph. Finally, outliers and influential observations were removed and model diagnostics were performed.

All model building was performed in the R Language and Environment for Statistical Computing (R Core Team (2014). R: A language and environment for statistical computing. R Foundation for Statistical Computing, Vienna, Austria. URL http://www.R-project.org/). Descriptive statistics were computed with STATISTICA (Data analysis software system, version 11. www.statsoft.com.) and are shown as median and interquartile range (IQR) while categorical variables are shown as n (%).

## Results

Of the 1372 people recruited, 325 did not present for testing, and a further 90 participants had missing clinical or laboratory data. The 957 participants included in this analysis consisted of 406 ART-naïve participants and 551 who had received ART for at least six months (ART-participants); 441 (81%) were on NNRTI-based therapy (ART1) while 107 (19%) were on PI-based therapy (ART2).

Baseline demographic and anthropometric variables are summarized in Table 1 and S1 and S2 Tables. The study cohort reflected the epidemiology of the HIV epidemic in South Africa and was predominantly female (78%) with a median age of 34 years (range 19–68 years). Median age at enrolment was higher in men than women (37.0 vs 33.0 years). Obesity (BMI ≥ 30 kg/m^2^) was more common than underweight and was found in 90/405 (22%) of naïve and 143/551 (26%) of treated participants. In the ART-naïve cohort 49/405 (12%) of participants were underweight (BMI < 20 kg/m^2^) while only 35/551 (6%) of participants receiving ART were underweight.

**Table 1:** Baseline demographic, anthropometric and clinical variables.

	Naïve		ART	
	Females	Males	Females	Males
Number (n)	309	97	435	116
Age (years)*	31 (27,37)	35 (31,41)	34 (29,40)	38 (33,45)
BMI (kg/m^2^)*	25.6(22.3,30.5)	22.1(20.3,24.2)	27.1(24.1,31.3)	22.6(20.6,25.1)
Systolic BP (mmHg)*	103(95,113)	110(100,122)	109(99,109)	117(107,128)
Diastolic BP (mmHg)*	68(60,77)	70(62,80)	72(64,79)	75(68,84)
Waist circumference (cm)*	81.0(74.0,91.3)	78.5(74.5,84.8)	87.3(79.5,97.0)	80.5(75.6,90.0)
Calf skin fold thickness (mm)*	17.8 (13.6,24.1)	6.1 (4.3,8.3)	16.8 (10.7,23.3)	5.7 (4.4,8.2)
CD4 count (cells/μl)*	278 (159,440)	227 (134,411)	371 (242,525)	278 (199,387)
Duration on treatment (months)*	NA	NA	13 (8,20)	13 (9,22)

*median (IQR), ART: Includes all participants receiving ART, NA: Not applicable.

### Lipids

**Fig 1:**
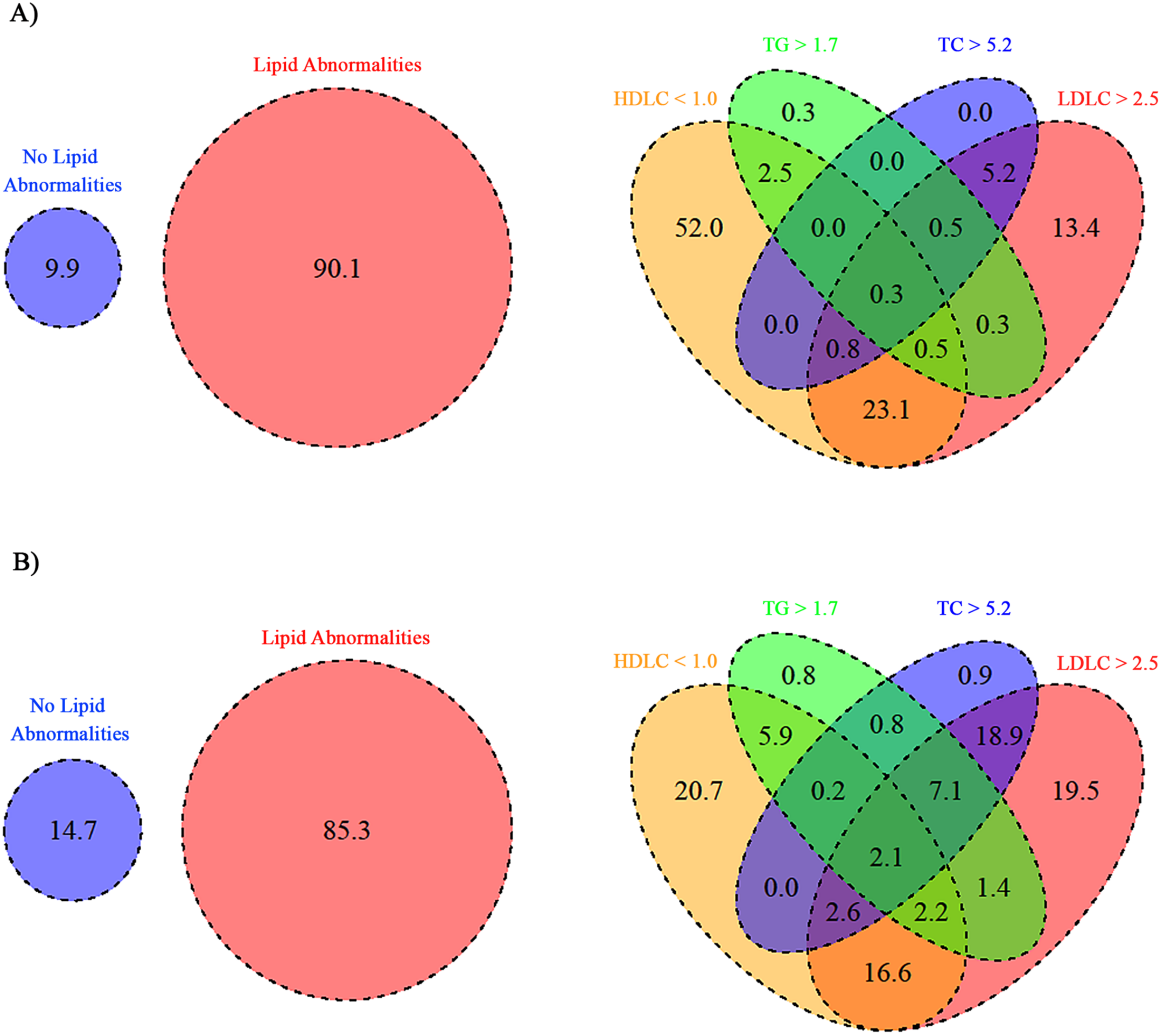
Distribution of lipid abnormalities in treatment naïve (A) and participants receiving ART (B). The left-sided Venn diagrams indicate % with dyslipidaemia. The right-sided diagrams indicate the individual lipid abnormalities in dyslipidaemic participants. Percentages in the right diagrams refer to % dyslipidaemic participants. Rounding accounts for percentages not equal to 100.

Baseline lipid values are summarized in Table 2, S3 Table and Fig 1. We classified 366/406 (90%) and 470/551 (85%) of ART-naïve and ART participants who had lipid results as dyslipidaemic using the definition given above (Fig 1). The most common lipid abnormality was low HDLC (<1 .0 mmol/L), found in 290/406 (71%) and 237/551 (43%) of ART naïve and ART participants respectively.

**Table 2:** Baseline lipid values.

	Naïve		ART	
	Females	Males	Females	Males
Number (n)	309	97	435	116
Total cholesterol (mmol/L)*	3.57(3.13,4.17)	3.77(3.38,4.37)	4.42(3.76,5.26)	4.41(3.81,5.44)
Triglycerides (mmol/L)*	0.84(0.66,1.11)	0.91(0.72,1.16)	0.98(0.77,1.39)	1.20(0.94,1.58)
HDLC (mmol/L)*	0.80(0.67,1.01)	0.92(0.73,1.10)	1.05(0.87,1.30)	1.09(0.83,1.45)
LDLC (mmol/L)*	2.32(1.93,2.81)	2.39(1.99,2.93)	2.80(2.22,3.40)	2.53(2.10,3.40)
Non-HDLC (mmol/L)*	2.70(2.35,3.24)	2.84(2.41,3.41)	3.30(2,70,3.98)	3.30(2.72,4.22)

*median (IQR), ART: Includes all participants receiving ART.

HDLC High density lipoprotein cholesterol;

LDLC Low density lipoprotein cholesterol

TC Total cholesterol

TG Triglycerides.

Severe hypercholesterolaemia, (TC > 7.5 mmol/L), was found in 0/406 (0%) and 6/551 (1%) of naïve and ART-treated participants, respectively. LDL hypercholesterolaemia of > 4.9 mmol/L was found in 1/406 (0.2%) naïve participants and 15/550 (2.7%) treated participants. Hypertriglyceridaemia >5.0 mmol/L was found in 0/406 (0%) naïve participants and 3/551 treated participants, whereas hypertriglyceridaemia >10 mmol/L was found in 1/406 (0.2%) naïve participants and 0/551 (0%) treated participants. Markedly low HDLC (<0.6 mmol/L) was found in 55/406(14%) and 22/551 (4%) of naïve and treated participants, respectively.

### Glycaemia

Detailed results on insulin resistance and glycaemic status have been reported previously. The glycaemic status of participants is summarized in Table 3.

**Table 3:** Glycaemic status based on OGTT.

	Naïve		ART	
	Females	Males	Females	Males
Normoglycaemia	249 (81%)	68 (70%)	327 (75%)	70 (60%)
Impaired fasting glucose	27 (9%)	16 (16%)	57 (13%)	29 (25%)
Impaired glucose tolerance	21 (7%)	11 (11%)	41 (9%)	11 (9%)
Diabetes	12 (4%)	2 (2%)	10 (2%)	6 (5%)

All data, n (%); Legend: Rounding accounts for percentages not equal to 100.

### Other cardiovascular risk factors

Current smoking was reported by 142/956 (15%) of participants but was almost 10 times more prevalent among men 104/212 (49%) than women 38/744 (5%). The mean ± SD systolic BP was 110 ± 17 mmHg with a diastolic BP of 72 ± 12 mm Hg. Hypertension (systolic BP ≥ 140 mmHg and/or a diastolic BP ≥ 90 mmHg), was present in 80/940 participants (9%) of whom the majority 57/80 (71%) were previously undiagnosed.

### ART-naïve participants

In ART-naïve participants we focussed our analysis on the effects CD4 count as a surrogate marker of immunosuppression on the lipid profile. Triglycerides and HDLC were both significantly associated with CD4 count, but not LDLC or TC.

Triglycerides correlated negatively with CD4 count (r = -0.196) ignoring potential confounders. Age, BMI and waist-hip ratio, but not gender and glycaemic status, were significantly associated with triglycerides. In the final multivariate model of triglycerides for ART-naïve participants (S4 Table), we found significant interaction effects between CD4 count and age (p < 0.001), and CD4 count and BMI (p < 0.001). For an average-aged patient with an average BMI, there was a significant negative correlation between triglycerides and CD4 count (p < 0.001). This relationship was attenuated in older participants and participants with higher BMIs. There was a significant positive relationship between waist-hip ratio and triglycerides (p < 0.001) independent of CD4 count.

HDLC was positively correlated (r = 0. 296) with CD4 count before adjusting for potential confounders. In our final model of HDLC (S5 Table), which included gender and glycaemic status as significant confounders, HDLC increased significantly more rapidly with CD4 count in diabetic compared to non-diabetic participants (p = 0.013), and HDLC was significantly higher in males compared to females irrespective of CD4 count (p = 0.009). Age, BMI and waist-hip ratio were not significant explanatory variables for HDLC after accounting for CD4 count, gender and glycaemic status.

### ART-treated participants

Ignoring potential confounders, triglycerides increased as calf skin fold thickness, a surrogate marker for lipoatrophy, decreased among the ART-treated participants (r = -0.204). The final multiple regression model (S6 Table) included BMI, waist-hip ratio, duration of stavudine exposure, efavirenz as the only NNRTI ever prescribed and NNRTI vs. PI-based ART as confounding factors in the relationship between triglycerides and calf skin fold thickness. Increased BMI and prolonged exposure to stavudine were associated with higher triglycerides (*p* = 0.025 and *p* < 0.001, respectively). The increase in triglycerides with decreasing calf skin fold thickness was greater for participants enrolled on PI-based therapy than for those on NNRTI-based therapy (*p* = 0.009) The relationship between calf skin fold thickness and triglycerides was attenuated in older participants (*p* = 0.017), holding WHR and ART treatment constant. We also found significant interactions between type of ART therapy and gender (*p* = 0.002), and type of ART therapy and exclusive efavirenz exposure (*p* = 0.008). For a female with the sample median calf skin fold thickness (13.1 mm), PI-based therapy was associated with higher triglycerides, though the effect was somewhat attenuated for a female who had only ever taken efavirenz as NNRTI. For a male with the sample median calf skin fold thickness, triglycerides were lower in participants receiving PI-based therapy if they had only ever taken efavirenz. In males who had been exposed to nevirapine, triglycerides were not affected by the type of ART. Calf skin fold thickness and ART type were not significant determinants of any other lipid variables in this study (see S7 and S8 Tables for the final models of HDLC and LDLC, respectively).

## Discussion

The major findings of our large cross-sectional study from a middle-income country include a very high prevalence of dyslipidaemia in both ART-naïve and treated participants, with low HDLC being by far the most common abnormality. We observed higher HDLC levels in participants receiving ART, suggestive of HDLC returning to baseline as participants improve clinically. Severe dyslipidaemia, defined here as LDLC >4.9 mmol/l or triglycerides >5.0 mmol/L, was an uncommon finding. Additionally, as previously reported, we found a high prevalence of dysglycaemia[13]. The association between untreated HIV infection and lipid abnormalities is well documented.[17–19] In most studies, HIV infection has been associated with low concentrations of HDLC, TC, LDLC and moderate hypertriglyceridaemia with a positive correlation between the degree of immunosuppression and lipid abnormalities. Chronic infection causes a persistent acute phase response, which typically is accompanied by an increase in triglycerides due to decreased lipoprotein lipase activity. An increase in triglyceride-rich lipoproteins and their remnants can promote exchange of triglycerides into HDL and subsequent lipolysis by hepatic lipase, accounting for lower HDLC concentration.[20, 21]

In our study, lipid abnormalities in ART-naïve participants are in agreement with previously reported studies.[22, 23] Increasing immunosuppression, as manifested by lower CD4 counts, correlated with an increase in triglycerides. Abdominal obesity, identified by an increase in waist-hip ratio (WHR), was associated with higher triglycerides values independent of CD4 count. Although triglycerides were inversely correlated with CD4 count, clinically important hypertriglyceridaemia was uncommon and was found in only 1/406 (0.2%) ART-naïve participant.

The most striking lipid abnormality in ART-naïve participants was markedly low HDLC, consistent with other studies from Nigeria and Uganda, but different from a rural Cameroonian population where only 18% of participants had a low HDLC with elevated triglycerides being the predominant lipid abnormality.[24–26] The median HDLC values of 0.80 mmol/L and 0.92 mmol/L for ART-naïve women and men, respectively are considerably lower than the mean female and male values of 1.28 mmol/L and 1.21 mmol/L, respectively reported for an urbanized black population in 1992, the caveat being that this was a relatively small study conducted more than twenty years ago.[27] Very low HDLC (<0.6 mmol/L) was present in 14% of ART-naïve participants. HDLC was correlated with CD4 count and was lower in participants with more advanced immunosuppression. Although ART-naïve women had a higher median CD4 count than men, their median HDLC was lower opposite to the usual gender differential observed for HDLC measurements. TC was lower than the previously reported population means of 4.4 mmol/L and 4.5 mmol/L for male and female urban black South Africans aged 30–44 years.[28] There are relatively few studies of dyslipidaemia in ART-naïve patients from lower and middle income countries (LMIC). In a recent review of studies from LMIC the lipid phenotype in ART-naïve participants was similar to that described in studies from high income countries (HIC), with elevated triglycerides, normal or reduced LDLC and low HDLC.[29]

The effects of ART on the lipid profile, glycaemia and body composition are well documented.[26, 30–35] In our study TC was higher in ART-treated participants than in treatment naïve participants, but was similar to previously described population averages.[28] The higher TC in ART-treated participants is likely due to both higher HDLC and LDLC values. Triglycerides increased with decreasing calf circumference as was shown in the study by Abrahams et al.[36] Increased BMI and prolonged exposure to stavudine were also positively associated with triglycerides. Importantly, calf circumference was not a significant determinant of any other lipid variable. Anthropometric differences between treatment naïve and ART groups were particularly marked in female participants. This is probably because HIV-negative black females have a larger BMI than their male counterparts resulting in greater changes in body composition during HIV-infection. Women on ART had higher BMI, waist circumference and WHR, but calf circumference was lower, probably due to exposure to stavudine. The observed differences in anthropometric parameters are suggestive of ART-associated lipodystrophy with an increase in visceral fat accompanied by loss of peripheral subcutaneous fat. There is substantial variation in the reported prevalence of lipodystrophy (7%-65%), associated with ART in low and middle income countries. [29]. Moreover, the prevalence of overweight and obesity was shown to increase in a South African study of patients on ART, adding to the cardiovascular and mortality risk. [37]

As PI-based therapy was only instituted when patients failed their first-line regimen, our cohort of participants receiving PI-based therapy was relatively small. Participants receiving ART2 were older and had longer ART exposure at study entry than participants receiving ART1. ART2 was associated with higher triglyceride concentrations, while there were only minor differences for the other lipid parameters. LPV/r has been documented to raise triglycerides and, to a lesser extent, cholesterol in previous studies.[38] [39]

Abnormal glucose metabolism was associated with higher triglycerides, TC, LDLC and non-HDLC in both ART-naïve and treated participants. Atherogenic dyslipidaemia characterized by moderate hypertriglyceridaemia, low HDLC and normal or modestly elevated LDLC, with small and dense LDL-particles, is common in diabetes but is also frequently found in patients with impaired glucose tolerance.[40, 41] Pre-diabetes is common among low and middle income countries in patients initiated on ART and studies from South Africa and Kenya indicate a prevalence of 23.5% and 16%, respectively.[13, 42]

Cardiovascular risk assessment algorithms such as the Framingham equation cannot be applied directly to our population, which differs significantly from the populations in which these algorithms were derived.[43] Assigning conventional risk increments to lipoprotein fraction changes, ART-treated patients would, on balance, likely have lower cardiovascular risk, due to their substantially increased HDLC in the face of a modest increase in TC resulting in a lower TC: HDLC ratio. It is, however, not certain whether the low concentrations of HDLC seen in untreated ART patients carry the same prognostic significance as low HDLC in otherwise well patients without chronic infection or inflammation. Furthermore HDLC is likely to increase in many treatment naïve patients once they are established on ART.[23, 32, 44] In our study, neither LDLC nor non-HDLC, as a measure of all atherogenic lipoproteins, was significantly higher in ART participants than previously reported population averages. The relatively low average age of our study cohort would suggest that short term (10 year) absolute cardiovascular risk is likely to be relatively low. The high rates of dysglycaemia in both naive and treated patients are of concern and may increase cardiovascular risk beyond that estimated by cardiovascular risk equations.

Our study has several important strengths and limitations. To the best of our knowledge this is one of the largest studies on the metabolic effects of ART from the African continent. Patients underwent a complete and detailed metabolic evaluation at study entry including detailed anthropometry, OGTT including measurement of insulin concentrations and fasting lipids. The size of our study allowed us to explore the effect of immunosuppression on serum lipid values in ART-naïve participants and the effects, if any, of body composition on lipid values in participants receiving ART. Additionally, we explored the impact of the specific ART regimen on lipid values. A large number of recruited patients did not present for testing, possibly partly explained by lack of access to transport, obtaining temporary work during the time of their appointment or illness keeping them house-bound. We did not assess any surrogate markers of atherosclerosis, such as carotid intima media thickness or coronary artery calcium score and we are thus unable to correlate biochemical abnormalities with evidence of subclinical atherosclerosis. We did not measure inflammatory markers such as C-reactive protein and thus, could not correlate inflammatory markers with metabolic abnormalities. Analysis of the metabolic changes associated with ART is furthermore constrained by the fact that the current study is cross-sectional. Due to the cross-sectional nature of the study, the nature and duration of ART are not uniform amongst participants. Similarly, there is substantial variation in the degree of immunological and clinical recovery amongst participants on ART. We do not have data from a control group of HIV uninfected people from the same community to compare metabolic parameters in a group that is likely to have a similar lifestyle and genetic background.

In conclusion we found that participants on ART had higher triglycerides, TC, LDLC and HDLC than those who were ART naïve. Although ART was associated with an increase in atherogenic lipoproteins, levels did not exceed previously reported population averages and in cardiovascular risk estimation are likely offset by the even greater increases in HDLC we observed. Severe lipid abnormalities requiring evaluation and treatment in the short term were uncommon in both treated and untreated participants. The absolute ten year cardiovascular risk is probably low in this cohort, as the majority of participants were female, relatively young and did not have markedly elevated concentrations of atherogenic lipoproteins. This assessment is, however, confounded by the lack of validated risk algorithms for patients with HIV infection, in whom multiple other factors such as chronic inflammation, endothelial damage and vascular inflammation may contribute to cardiovascular risk.

## Supporting Information

S1 Dataset Data used to generate results in this manuscript. (XLSX)

S1 Table Additional baseline clinical, demographic and anthropometric variables. (DOCX)

S2 Table Baseline demographic and anthropometric variables by ART category. (DOCX)

S3 Table Lipid values by ART category. (DOCX)

S4 Table Regression model of log(triglycerides) for ART-naïve participants. (DOCX)

S5 Table Regression model of log(HDLC) for ART-naïve participants. (DOCX)

S6 Table Regression model of log(triglycerides) for participants on ART. (DOCX)

S7 Table Regression model of log(HDLC) for participants on ART. (DOCX)

S8 Table Regression model of log(LDLC) for participants on ART. (DOCX)
