# Supplementary material for: Anti-Retroviral Therapy Increases the Prevalence of Dyslipidemia in South African HIV-Infected Patients
Source: PLoS One. 2016 Mar 17;11(3):e0151911. doi: 10.1371/journal.pone.0151911 (PMC4795704; doi:10.1371/journal.pone.0151911)
Supplement: S1 Table — (DOCX) [file pone.0151911.s002.docx]

S1 Table: Additional baseline clinical, demographic and anthropometric variables

| Number (n) | | **Naïve** | | **ART** | |
| --- | --- | --- | --- | --- | --- |
|  |  | Females | Males | Females | Males |
|  |  | 309 | 97 | 435 | 116 |
| Education n,(%) | None | 12 (4%) | 13 (13%) | 25 (6%) | 7 (6%) |
|  | Primary | 48 (16%) | 26 (27%) | 58 (13%) | 34 (29%) |
|  | Secondary | 239 (77%) | 56 (58%) | 340 (78%) | 71 (61%) |
|  | Tertiary | 10 (3%) | 1 (1%) | 12 (3%) | 4 (3%) |
| Smoking n, (%) ^1^ |  | 21 (7%) | 49 (51%) | 17 (4%) | 55 (47%) |
| Alcohol n, (%) ^2^ |  | 96 (31%) | 60 (62%) | 75 (17%) | 48 (41%) |

Legend: Rounding accounts for percentages not equal to 100

^1^ Current smoker

^2^ Consumed alcohol in the last 12 months
