# Supplementary material for: Anti-Retroviral Therapy Increases the Prevalence of Dyslipidemia in South African HIV-Infected Patients
Source: PLoS One. 2016 Mar 17;11(3):e0151911. doi: 10.1371/journal.pone.0151911 (PMC4795704; doi:10.1371/journal.pone.0151911)
Supplement: S2 Table — (DOCX) [file pone.0151911.s003.docx]

S2 Table: Baseline demographic and anthropometric variables by ART category

|  | **ART1** | | **ART2** | |
| --- | --- | --- | --- | --- |
|  | Females | Males | Females | Males |
| Number (n) | 345 | 99 | 90 | 17 |
| Age (years)* | 33.0(29.0, 40.0) | 37.0(33.3, 45.0) | 35.0(30.0, 40.0) | 41.0(35.0, 48.0) |
| BMI (kg/m^2^)* | 27.1(24.2, 31.2) | 22.4(20.6, 25.1) | 26.6(24.1, 33.3) | 23.1 (20.0, 24.3) |
| Waist circumference (cm)* | 87.0(79.3, 96.5) | 80.3(75.0, 90.0) | 89.3(81.0, 100.0) | 83.0(77.3, 90.0) |
| Calf skin fold thickness (mm)* | 16.6(10.7, 22.4) | 5.8(4.4, 8.4) | 17.5(11.6, 25.0) | 4.4(4.0, 6.8) |
| CD4 count (cells/µl)* | 335(227, 479) | 279(204, 387) | 494(312, 663) | 247(142, 467) |
| Duration on treatment (months)* | 16.0(10.0, 25.0) | 13.0(9.0, 23.0) | 33.0(25.5, 47.0) | 29.0(23.0, 40.0) |
| Duration of D4T exposure (months)* | 13.0(8.0, 19.0) | 13.0(9.0, 22.0) | 14.0(10.0, 23.0) | 18.5(10.5, 23.5) |

*Median (IQR)

ART1: NNRTI-based ART

ART2: PI-based ART
