# Supplementary material for: Anti-Retroviral Therapy Increases the Prevalence of Dyslipidemia in South African HIV-Infected Patients
Source: PLoS One. 2016 Mar 17;11(3):e0151911. doi: 10.1371/journal.pone.0151911 (PMC4795704; doi:10.1371/journal.pone.0151911)
Supplement: S3 Table — (DOCX) [file pone.0151911.s004.docx]

S3 Table: Lipid values by ART category

|  | **ART 1** | | **ART 2** | |
| --- | --- | --- | --- | --- |
|  | Females | Males | Females | Males |
| Number (n) | 345 | 99 | 90 | 17 |
| Total cholesterol (mmol/L)* | 4.49(3.80, 5.27) | 4.38(3.74, 5.48) | 4.32(3.65, 5.25) | 4.58(3.99, 5.41) |
| Triglycerides (mmol/L)* | 0.94(0.74, 1.30) | 1.18(0.91, 1.55) | 1.28(0.89, 1.81) | 1.33(1.20, 1.81) |
| HDLC (mmol/L)* | 1.07(0.89, 1.33) | 1.11(0.83, 1.49) | 0.99(0.79, 1.18) | 1.03(0.82, 1.29) |
| LDLC (mmol/L)* | 2.83(2.28, 3.47) | 2.48(2.00, 3.43) | 2.60(2.09, 3.19) | 2.62(2.24, 3.37) |
| Non-HDLC (mmol/L)* | 3.30(2.70, 3.98) | 3.12(2.61, 4.24) | 3.29(2.66, 3.97) | 3.35(3.22, 4.21) |

*Median (IQR)

ART1: NNRTI based ART

ART2: PI based ART
