# Supplementary material for: Anti-Retroviral Therapy Increases the Prevalence of Dyslipidemia in South African HIV-Infected Patients
Source: PLoS One. 2016 Mar 17;11(3):e0151911. doi: 10.1371/journal.pone.0151911 (PMC4795704; doi:10.1371/journal.pone.0151911)
Supplement: S4 Table — (DOCX) [file pone.0151911.s005.docx]

S4 Table: Regression model of log(triglycerides) for ART-naïve participants

|  | Coefficient | Standard Error | *p*-value |
| --- | --- | --- | --- |
| Intercept | -8.798 × 10^-1^ | 2.610 × 10^-1^ | < 0.001 |
| CD4 count | -2.496 × 10^-3^ | 4.200 × 10^-4^ | < 0.001 |
| Age | 1.045 × 10^-4^ | 3.271 × 10^-3^ | 0.975 |
| BMI | -1.205 × 10^-2^ | 4.804 × 10^-3^ | 0.013 |
| Waist-Hip Ratio | 1.405 | 2.612 × 10^-1^ | < 0.001 |
| CD4/Age Interaction | 2.526 × 10^-5^ | 8.417 × 10^-6^ | 0.003 |
| CD4/BMI Interaction | 4.636 × 10^-5^ | 1.028 × 10^-5^ | < 0.001 |
