# Supplementary material for: Anti-Retroviral Therapy Increases the Prevalence of Dyslipidemia in South African HIV-Infected Patients
Source: PLoS One. 2016 Mar 17;11(3):e0151911. doi: 10.1371/journal.pone.0151911 (PMC4795704; doi:10.1371/journal.pone.0151911)
Supplement: S5 Table — (DOCX) [file pone.0151911.s006.docx]

S5 Table: Regression model of log(HDLC) for ART-naïve participants

|  | Coefficient | Standard Error | *p*-value |
| --- | --- | --- | --- |
| Intercept | -0.348 | 0.032 | < 0.001 |
| CD4 count | 4.176 × 10^-4^ | 7.936 × 10^-5^ | < 0.001 |
| Male | 0.107 | 0.041 | 0.009 |
| Diabetic | -0.336 | 0.182 | 0.065 |
| CD4/Diabetic interaction | 0.001 | 4.527 × 10^-4^ | 0.013 |
