# Supplementary material for: Anti-Retroviral Therapy Increases the Prevalence of Dyslipidemia in South African HIV-Infected Patients
Source: PLoS One. 2016 Mar 17;11(3):e0151911. doi: 10.1371/journal.pone.0151911 (PMC4795704; doi:10.1371/journal.pone.0151911)
Supplement: S6 Table — (DOCX) [file pone.0151911.s007.docx]

S6 Table: Regression model of log(triglycerides) for participants on ART

|  | Coefficient | Standard Error | *p*-value |
| --- | --- | --- | --- |
| Intercept | -2.352 | 0.440 | < 0.001 |
|  |  |  |  |
| **Main effects** |  |  |  |
| Only efavirenz | 0.161 | 0.041 | < 0.001 |
| Stavudine duration | 0.007 | 0.002 | < 0.001 |
| BMI | 0.010 | 0.005 | 0.025 |
| Age | -7.747 × 10^-5^ | 0.004 | 0.985 |
| Male | 0.065 | 0.055 | 0.241 |
| Waist-hip ratio | 2.294 | 0.537 | < 0.001 |
| PI-based ART | 0.663 | 0.131 | < 0.001 |
| Calf skin fold thickness | 0.018 | 0.025 | 0.467 |
|  |  |  |  |
| **Interaction effects** |  |  |  |
| ART2/Only efavirenz | -0.249 | 0.093 | 0.008 |
| ART2/Male | -0.428 | 0.135 | 0.002 |
| Calf/Age | 5.950 × 10^-4^ | 2.490 × 10^-4^ | 0.017 |
| Calf/Waist-hip ratio | -0.050 | 0.029 | 0.087 |
| Calf/PI-based ART | -0.016 | 0.006 | 0.009 |
