# Supplementary material for: Anti-Retroviral Therapy Increases the Prevalence of Dyslipidemia in South African HIV-Infected Patients
Source: PLoS One. 2016 Mar 17;11(3):e0151911. doi: 10.1371/journal.pone.0151911 (PMC4795704; doi:10.1371/journal.pone.0151911)
Supplement: S8 Table — (DOCX) [file pone.0151911.s009.docx]

S8 Table: Regression model of log(LDLC) for participants on ART

|  | Coefficient | Standard Error | *p*-value |
| --- | --- | --- | --- |
| Intercept | 0.833 | 0.068 | < 0.001 |
| Age | 0.004 | 0.002 | 0.023 |
